# A LoRa-Based Mesh Network for Peer-to-Peer Long-Range Communication

**DOI:** 10.3390/s21134314

**Published:** 2021-06-24

**Authors:** Riccardo Berto, Paolo Napoletano, Marco Savi

**Affiliations:** Department of Computer Science, Systems and Communications, University of Milano-Bicocca, 20126 Milan, Italy; riccardo.berto@unimib.it (R.B.); marco.savi@unimib.it (M.S.)

**Keywords:** LoRa, ad hoc networks, mesh networking, peer-to-peer networking

## Abstract

LoRa is a long-range and low-power radio technology largely employed in Internet of Things (IoT) scenarios. It defines the lower physical layer while other protocols, such as LoRaWAN, define the upper layers of the network. A LoRaWAN network assumes a star topology where each of the nodes communicates with multiple gateways which, in turn, forward the collected data to a network server. The main LoRaWAN characteristic is the central role of the gateways; however, in some application scenarios, a much lighter protocol stack, relying only on node capabilities and without the presence of gateways, can be more suitable. In this paper, we present a preliminary study for realizing a LoRa-based mesh network, not relying on LoRaWAN, that implements a peer-to-peer communication between nodes, without the use of gateways, and extends node reachability through multi-hop communication. To validate our investigations, we present a hardware/software prototype based on low-power-consumption devices, and we preliminarily assess the proposed solution.

## 1. Introduction and Background

The Internet of Things (IoT) has become an essential and pervasive means in our society. Nowadays, more and more industrial, commercial and customer applications rely on data collected by a multitude of heterogeneous devices located at the extreme borders of the network. Given their high density, their limited battery availability and their inaccessibility in most of the cases, the research community has struggled to design lightweight solutions to ensure long-range, low-power and low-bitrate wireless transmissions to/from those devices, opening the door to the deployment of the so-called LPWANs (low-power wide-area networks) [1].

A well-known wireless technology for LPWANs is LoRa (long range) [2]. LoRa implements a physical layer that combines the chirp spread spectrum (CSS) radio modulation with integrated forward error correction (FEC) for enabling robust long-range communications on unlicensed industrial, scientific and medical (ISM) frequency bands. Given its robustness and versatility, LoRa has quickly become the most widely adopted physical layer for LPWANs. Concerning the upper layers, the LoRa Alliance has then proposed LoRaWAN [3], an open media access control (MAC) and network protocol that allows LoRa-based devices to communicate and that inherently adopts a well-defined network architecture [4].

A LoRaWAN network includes three architectural components: the end devices, the gateways and a remote network server. These components are inter-connected in a “star-of-stars” topology, where end devices communicate with one or more gateways (using LoRa as the physical layer) and where each gateway dispatches LoRaWAN frames to the network server using a higher-throughput backhaul interface (e.g., WiFi or 5G). Then, applications interfacing with the server can make the best use of the collected data. LoRaWAN is the most widely adopted L2/L3 protocol for LPWANs, although some limitations have been identified [5,6].

One of the strongest limitations of LoRaWAN is the adopted topology, where only direct single-hop communication is allowed between end devices and gateways. Even though this configuration is suitable for many applications, in some cases (e.g., when data must be gathered/exchanged from/in difficult-to-access areas) it is far from being the optimal solution. Many works in the literature have then dealt with enabling *mesh* networking and multi-hop communication in LoRaWAN or on similar alternative LoRa-based architectures, where end devices can act as relay nodes, to extend network coverage and improve energy consumption [7].

Even though a significant step forward has been made by these works, we believe that it is just an intermediate step. In fact, all of them still partially embrace a LoraWAN or LoRaWAN-like network architecture, and extend it towards supporting a “star-of-meshes” topology. This means that gateways still play a central role as concentrators, and that data need to be finally conveyed through Internet/broadband access, to a remote location before being made accessible to applications. This is clearly not ideal in application scenarios where it would be better to keep data local for privacy or performance reasons (e.g., in the case of emergency applications for disaster recovery or first responders support). Additionally, also in the case of a privacy-preserving and high-speed private network infrastructure where data collected by the gateway are not conveyed through the public Internet but through dedicated backhaul links, the gateway is a single point of failure whose malfunctioning would compromise the operation of the whole LPWAN network.

In this paper we pave the way towards filling this gap. We propose and preliminarily evaluate a hardware/software LoRa-based solution that, being completely gateway-free, enables peer-to-peer communication among LoRa end devices, while also preserving multi-hop and mesh networking functionalities as proposed in previous works. The solution, developed on top of the LoRa physical layer, is meant to provide a lighter network stack than LoRaWAN, so that low-cost, flexible and easy-to-configure “out-of-Internet” communication can be ensured wherever and whenever needed.

Our proposal is extremely cheap (no LoRaWAN gateway needs to be bought and configured) and effective: each of the end devices acts as a simplified gateway, which can be accessed through its USB serial port by a more powerful device (e.g., a laptop). In this case, the limited computational capacity of the single node can be increased in order to embrace more computational demanding application scenarios. Our preliminary results have shown how, by exploiting different LoRa transmission setups (i.e., modulations), it is possible to strike the most desirable balance between network coverage and end-to-end transmission delay.

The remainder of this paper is structured as follows. Section 2 recalls the related work. Section 3 describes the proposed architecture, while Section 4 summarizes our preliminary results. Finally, Section 5 concludes the paper and reports on the planned future work.

## 2. Related Work

In this section we recall the related work that can be found in literature and to what extent our work overcomes the state-of-the-art approaches. We first focus on existing solutions for mesh and multi-hop networking in LoRa-based networks, then we report some relevant use cases.

### 2.1. LoRa-Based Mesh and Multi-Hop Networking

One of the first works exploring LoRa capabilities to build a generic IoT mesh and multi-hop networks is Reference [8]. The paper proposes LoraBlink, a protocol supporting multi-hop communication. The adopted architecture includes multiple LoRa-based nodes and a sink, which is the final destination of all the messages generated by the nodes. A similar approach is proposed in References [9,10]. In these papers it is shown that the packet delivery ratio can be improved by mesh networking with respect to simple star-topology configurations, and the sink is explicitly called a gateway, adopting a similar nomenclature as the one used for LoRaWAN. Similarly, Reference [11] demonstrates the capabilities of LoRa-based mesh/multi-hop networking in a city-wide testbed. Further, in this case, the gateway plays a crucial role, being in charge of orchestrating the communication with the LoRa-based nodes using a polling mechanism. A similar architecture is proposed in Reference [12], but it is based on time-slotted event-driven communication to keep collision rates low.

Even though we take inspiration from all of these works to design a solution ensuring mesh networking capabilities and multi-hop communication, we eliminate the need of defining a *nodes’ hierarchy*, where a node with enhanced functionalities (i.e., the gateway) is needed. We instead propose a flat and peer-to-peer solution, where all the nodes (or none of them) can be seen as gateways that can be exploited as access points to external networks (e.g., Internet). The only work that we found proposing a similar peer-to-peer architecture is Reference [13]; however, it focuses on the definition of a stand-alone Lora-based IoT network, and the possibility to interface the LoRa-based nodes to external networks is not explored.

### 2.2. Use Cases

Some works focus on specific use cases of the architectures discussed above, including forest fire detection [14], wild animals [15] and livestock [16] tracking, urban drainage [17] and monitoring of underground environments (e.g., medieval aqueducts) [18]. In all these cases, remote areas can be reached by multi-hop communication and/or mesh networking capabilities, and collected data are finally conveyed towards a gateway for further routing and processing.

However, the gateway is a single point of failure that, for some specific use cases, should be avoided. This is especially true when the LoRa-based nodes need to be used in emergency situations to monitor, e.g., natural disasters such as fires or floodings, where the environment dynamically changes over time and where some areas (including the one where the gateway is placed) can quickly become inaccessible. In this specific scenarios, a set of cheap LoRa-based nodes (as the ones adopted in this paper) can be spread throughout the territory, accepting the risk that they may be lost. Our gateway-free solution, where each node can potentially be accessed by external networks, assures constant accessibility to the mesh network and avoids the presence of a single point of failure, being thus more flexible.

## 3. System Architecture

In this section, we report the proposed layered system architecture for mesh, multi-hop and peer-to-peer networking, including both design and high-level implementation choices.

### 3.1. Network Design and Configuration

**Figure 1:**
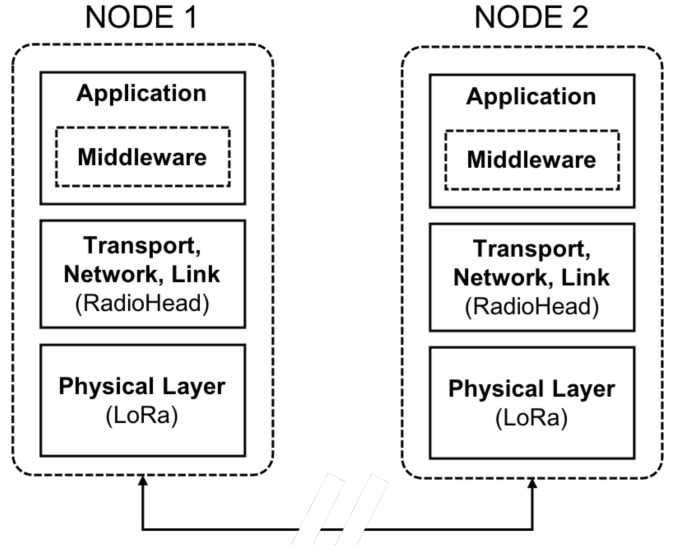
Proposed LoRa-based stack for peer-to-peer communication.

The network stack proposed here considers three layers (see Figure 1): *(i)* a physical layer based on the standard LoRa communication protocol; *(ii)* a link, network and transport layer for addressing, routing and meshing; *(iii)* an application layer as interface with real applications (possibly accessible by external networks), including a middleware that enqueues and assigns priorities to the messages that need to be aired. The first two layers are based on a public library designed for embedded microprocessors, named RadioHead (https://www.airspayce.com/mikem/arduino/RadioHead/ (accessed on 22 June 2021)).

**Figure 2:**
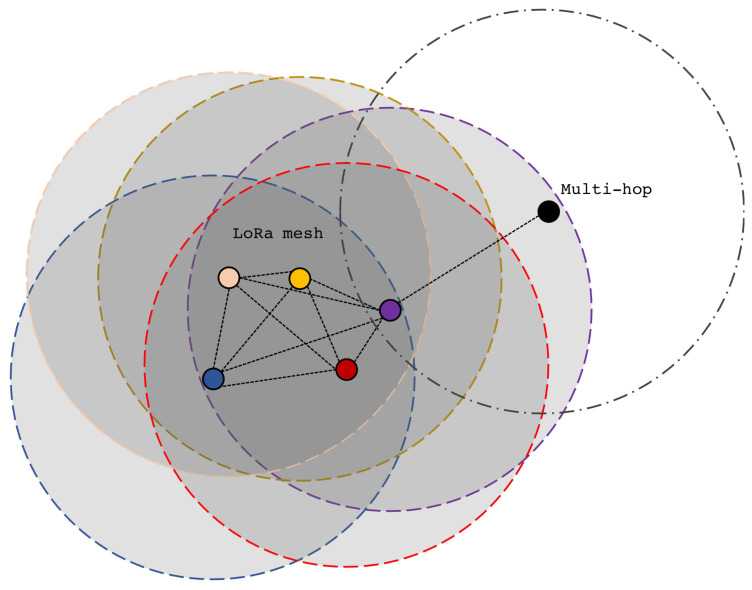
Peer-to-peer LoRa-based mesh network with multi-hop capabilities.

Our proposed LoRa-based network envisions a fully connected topology that permits peer-to-peer communication between end devices (from now on simply called nodes) only if their distance is less than *D* (see the dotted circles of Figure 2). Nodes at a distance higher than *D* can communicate by exploiting *multi-hop* communication if they are connected to other nodes that can convey the message (see the violet and the black nodes of Figure 2), enhancing end-to-end node reachability. Clearly, each node embeds the aforementioned network stack.

**Figure 3:**
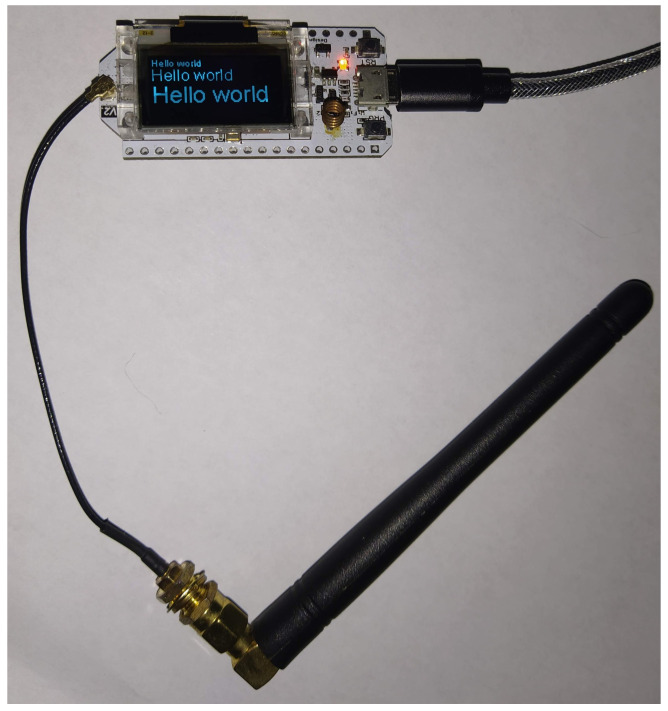
One of the nodes of the network, complete with its antenna.

Nodes are low-cost, low-power systems based on a microcontroller board embedding a LoRa chip. For our implementation we use the *ESP32 Heltec WiFi LoRa V2* board (https://heltec.org/project/wifi-lora-32/ (accessed on 22 June 2021)), which comes with the *Semtech SX1276* LoRa transceiver (https://www.semtech.com/products/wireless-rf/lora-transceivers/sx1276 (accessed on 22 June 2021)) and costs around USD 20. Figure 3 shows a picture of the *ESP32 Heltec WiFi LoRa V2* along with its external antenna, whose gain is 3 dBi.

Moreover, given our hardware choice, the nodes of the network have adequate processing capabilities to enable real applications (e.g., exchange of text messages and exchange of collected sensors’ data) to run on the board. Additionally, the nodes are small and lightweight enough to be adopted in diverse real-world scenarios: for instance, they could be mounted on mobile devices, such as unmanned vehicles, so that dynamic and mobile mesh networks can be created.

### 3.2. Physical Layer

The low-level access to the media channel is guaranteed by the RadioHead library, which interacts with the chosen LoRa chip. The library supports LoRa with frequency hopping spread spectrum (LR-FHSS) and provides access to different transmission modulations (i.e., *setups*). More details on supported and assessed setups are given in Section 4.

### 3.3. Link, Network and Transport Layer

Node *addressing* follows the specifications of the RadioHead library: 8-bit identifiers are adopted, meaning that a maximum number of $2^{8}-1$ nodes can be addressed, since the 255 address is used for broadcast transmission mode. For each node, a *matrix-based routing table* is stored in memory: the relatively limited number of addressable nodes is strictly related to the use of such table and to the board’s memory size. Increasing the number of addressable nodes (e.g., $2^{16}$ or $2^{32}$), would require larger memory, which is not compatible with the low-power-consumption electronics adopted in our implementation. A custom library specifying a different addressing strategy or more sophisticated strategies to store only relevant addresses, such as the one proposed in [19], could be designed to overcome this limitation, but this is out of the scope of this paper.

The RadioHead library also specifies a custom 4-bytes header (including *to*, *from*, *sequence number* and *flags* fields). More in detail, the RadioHead library documentation reports the following: “*Each message sent and received by a RadioHead driver includes 4 headers: (1) TO—The node address that the message is being sent to (broadcast RH_BROADCAST_ADDRESS (255) is permitted); (2) FROM—The node address of the sending node; (3) ID—A message ID, distinct (over short time scales) for each message sent by a particular node; (4) FLAGS—A bitmask of flags. The most significant 4 bits are reserved for use by RadioHead. The least significant 4 bits are reserved for applications.*” The header includes the information for the *routing* and *forwarding* of unreliable (or reliable through retransmission, upon request) variable-length messages in the form of *datagrams*, with an optional broadcast mode, as already specified above. Multi-hop delivery of datagrams from a source to a destination node is guaranteed via zero or more intermediate nodes, with automatic route discovery and re-discovery by means of special *route discovery request* broadcast packets, generated by the source and conveyed to the destination, which are followed by a unicast *route discovery response*.

## 4. Experimental Evaluation

In this section we provide some details on the adopted hardware equipment, including its configuration, and we report our experimental and numerical evaluation. We first focus on delivery time assessment and single-node transmission efficiency in the presence of multiple messages to be aired/received. We then present our evaluation single and multi-hop communication efficiency and delivery times.

### 4.1. Hardware Equipment and Transmission Setups

The adopted ESP32 controller (see Figure 3) offers a micro USB port for serial communications, a 0.96 inch OLED screen, a JST 1.25 2-pin battery connector and, as already said, an on-board SX1276 LoRa transceiver; it comes with a Li-ion battery management circuitry that eases the deployment when working with LoRa. The board is particularly suitable for mesh networking because it has a good computational capability and very low power consumption; it is equipped with a two-cores Xtensa CPU (https://www.espressif.com/sites/default/files/documentation/esp32_datasheet_en.pdf (accessed on 22 June 2021)) with 512 KB of RAM and a 4 MB on-board flash memory. The micro USB serial port can be used to connect the board to more powerful data sources (e.g., a laptop or a smartphone) or to sensors for data gathering.

The RadioHead library supports several transmission setups on the SX1276 chip that differ in terms of bandwidth (BW), coding rate (CR) and spreading factor (SF): the CR refers to the proportion of transmitted bits that actually carry information, the SF is the number of bits per symbol, while BW refers to the difference between the upper and lower frequencies occupied by the chirp. In spread-spectrum modulation techniques, a chirp is a sinusoidal signal with increasing frequency over time. Table 1 reports the transmission setups considered in this paper, compliant with EU regulations. In all the cases CR is set to 4/5, while BW and SF vary. Among all the possible transmission setups that could be chosen by varying BW and SF, we chose those that make it possible to best explore the different trade-offs investigated in this paper, as we demonstrate in the following section.

**Table 1:** Transmission setups and delivery time (with 95% confidence interval) for single-hop communication between A and B (Figure 4) and two-hop communication between A and C (Figure 5).

			Delivery Time (ms)	
Transmission Setup	BW (KHz)	SF	Single-Hop	Two-Hop
BroadBand	250	7	524±93	863±109
NarrowBand	125	7	678±202	1362±430
NarrowBand+	125	11	5358±57	10,774±91
NarrowBand++	125	12	9315±56	18,636±308
VeryNarrowBand	31.25	9	6721±95	13,438±229

Since the employed controller only permits half-duplex communication, the overall transceiver system should be designed to spend as much time as possible in an active listen state so that expensive retransmissions, due to missing payloads or acknowledgments (ACKs), are avoided. This is guaranteed by a carefully weighted software solution that leverages both the cores of the ESP32 Xtensa CPU. As also shown in Figure 6, a core is dedicated to handling the SX1276 LoRa transceiver (*network core* or *Core0*), while the other one is left to applications’ use (*application core* or *Core1*). The former core is also entitled for handling the JSON encoding and decoding of the aired payloads, so that the entire network stack is kept out of the application core. As shown in Section 4.3, in our tests, the application core runs a simple web server; however, nothing prevents us from running some custom logic implementing a different and even more complex distributed application, depending on the target application scenario. Since messages between cores are exchanged using FreeRTOS (https://freertos.org/ (accessed on 22 June 2021)) synchronization primitives, where a thread-safe unbounded FreeRTOS queue is employed, the application core constructs a string pointer and pushes it to the multicore-aware queue along with a recipient address. The network core then takes care of airing the enqueued message and ensures that it is delivered to the right node.

### 4.2. Delivery Time Assessment

As a first evaluation, we assessed in a lab the delivery times in the case of single or two-hop communication in the LoRa-based mesh network.

#### 4.2.1. Experimental Settings

**Figure 4:**
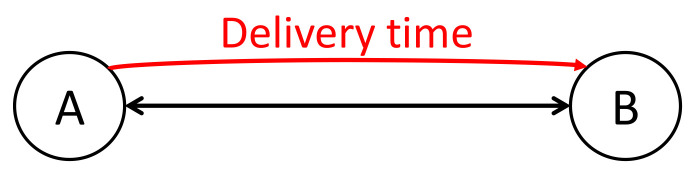
Single-hop delivery time evaluation (Table 1).

**Figure 5:**
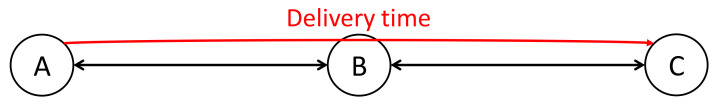
Two-hop delivery time evaluation (Table 1).

For each transmission setup, we evaluated the delivery time when a 240-bytes payload message is sent. The maximum payload that can be sent by the SX1276 transceiver is 255 bytes, including 4 bytes of the RadioHead header: this means that the maximum net payload size that can be transmitted is 251 bytes. We have chosen a smaller message size of 240 bytes (244 if the RadioHead header is included in the count) so that it can be ensured that an (up to) 11-bytes nonce can be appended to the header in the case of payload ciphering, which we did not perform in our experiments. This choice puts us in the worst-case scenario in terms of message size. In these experiments we relied on one-way transmissions. Figure 4 and Figure 5 show the network setup, where up to three nodes (A, B and C) belong to the LoRa-based mesh. We considered single-hop and two-hop communication as shown in those figures. In the case of two-hop communication, the node B is able to correctly route traffic between A and C nodes. The computed delivery time refers to the difference between the time when the last byte of the message was correctly elaborated by node B or C (depending on the test) at the application layer and the time when the application layer at node A generated the message to be aired. We ensured that nodes clock times were synchronized. Propagation times can be considered negligible.

#### 4.2.2. Results

Table 1 shows the results of this evaluation. For each delivery time value, we report the average value along with its 95% confidence interval. Since propagation times are negligible, only physical transmission/reception (through LoRa transceivers) and elaboration/routing times on the ESP32 boards are assessed with these experiments. It is clear that SF plays a key role: the higher the SF is, the higher the delivery time. Another important consideration is that the delivery time for two-hop communication is around twice the delivery time for single-hop communication in all the cases. This means that the routing/elaboration time in the intermediate node is almost negligible and that the overall delivery time is dominated by the time needed to send/receive the message through the LoRa transceivers. We thus expect that the delivery time, in the case of an *m*-hop communication with m>2, will be around *m* times the delivery time of the single-hop case (see Section 4.4).

### 4.3. Single-Node Transmission Efficiency Assessment

We then emulated and assessed, in a lab, a typical load condition of a node: multiple messages generation (or sensor readings) and their subsequent transmission on the LoRa-based mesh network.

#### 4.3.1. Experimental Setting

**Figure 6:**
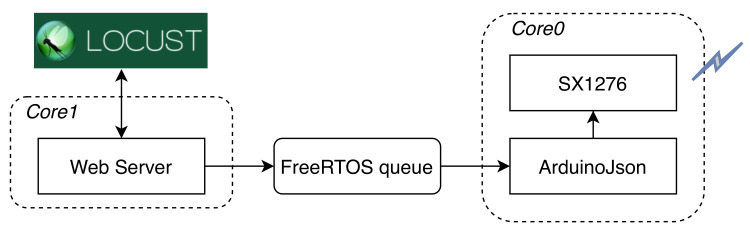
Experimental configuration for transmission efficiency assessment.

We used the Locust HTTP testing framework (https://locust.io/ (accessed on 22 June 2021)) to produce multiple HTTP requests from an external IP network to an HTTP Web Server running on the application layer of the node. Figure 6 shows the experimental configuration: the Locust software, running on an external device, makes a request including the 240-bytes payload message; the web server sends a *200 OK* message back to Locust if the message has been successfully forwarded to the mesh through LoRa, or a *500 InternalServerError* otherwise. As explained in Section 4.1, we exploited the dual-core-based architecture of the ESP32. The HTTP web server runs on *Core1* (we used the Arduino-ESP32 standard library (https://github.com/espressif/arduino-esp32 (accessed on 22 June 2021)) to implement it), while the JSON parsing and the transmission operations run on *Core0*. To alleviate the load of *Core0*, we adopted a FreeRTOS queue with variable size to enqueue messages coming from *Core1* to be transmitted. We configured Locust to send 1, 3 and 5 parallel requests at different times, with FreeRTOS queue size ranging from 1 to 20, and we evaluated the ratio of requests per second that are successfully handled by the node (i.e., which generate a *200 OK*) against the number of Locust-generated requests in the same time frame: we call this metric *transmission efficiency*. The NarrowBand transmission setup, which is the default one in the RadioHead library, was adopted.

#### 4.3.2. Results

**Figure 7:**
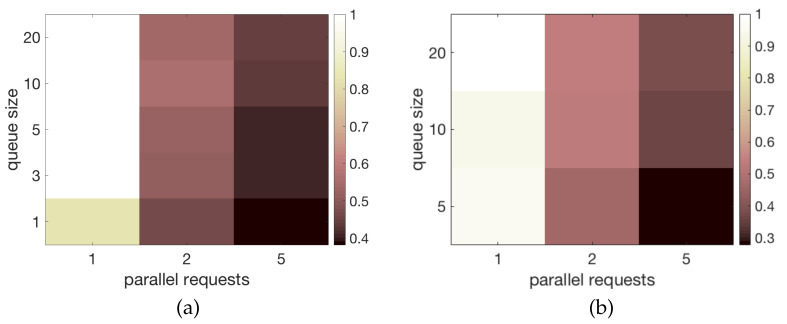
Transmission efficiency with different FreeRTOS queue sizes. (**a**) Without acknowledgment. (**b**) With acknowledgment.

Figure 7a shows the transmission efficiency when no acknowledgment is required. A single request per second can be handled with a very high efficiency (i.e., higher than 0.8) with any queue size. Two parallel requests per second can be handled with an efficiency of about 0.6 with a queue size of at least 3, while five parallel requests per second are always handled with a very low efficiency, below 0.4. Figure 7b shows the transmission efficiency when an acknowledgment is required for each aired message. A single request per second can be handled with a very high efficiency (i.e., higher than 0.9) with any queue size. Two parallel requests per second can be handled with a good efficiency, higher than 0.6 with a queue size of at least 5. Five parallel requests per second are instead always handled with a very low efficiency, below 0.4 with a a queue size of at least 5. These results show that the adopted hardware is suitable for applications that require few parallel transmissions, as it happens in most IoT scenarios.

### 4.4. Single and Multi-Hop Performance Assessment

In our last evaluation, we measured the single-hop *communication efficiency* between two nodes placed at a variable distance, defined as the ratio between the number of correctly received messages against the total number of sent messages. We also numerically evaluated the expected performance in the case of multi-hop communication.

#### 4.4.1. Experimental Setting

**Figure 8:**
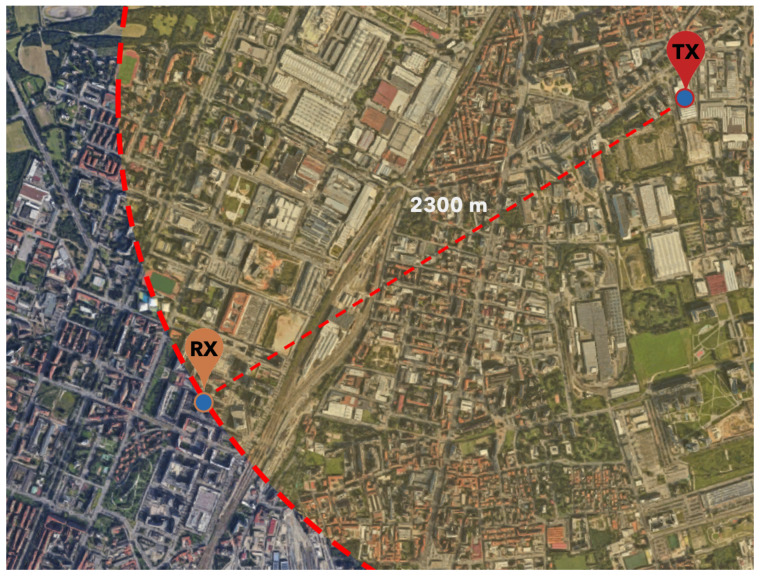
Urbanization of the northern Milan area, where the test took place.

The receiver was placed near to the window of a room at the 6th floor of a building while the transmitter moved in the surrounding area, covering a distance ranging from 400 to 2300 m. Figure 8 illustrates a Google Maps Satellite glimpse of the environment, showing the urbanization level where the test took place; all the transmission setups were tested.

#### 4.4.2. Results

**Figure 9:**
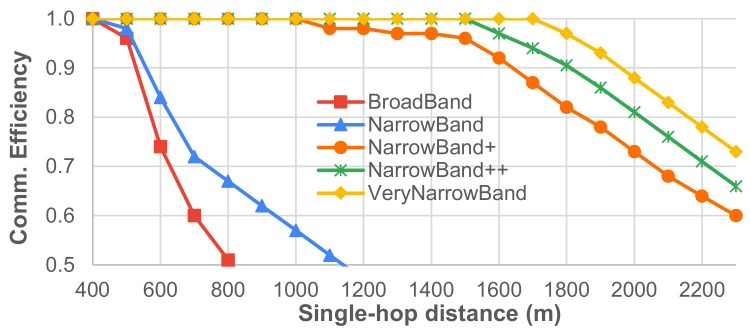
*Single-hop* communication efficiency at different distances.

Figure 9 shows that the various transmission setups lead to very different single-hop communication efficiencies. A clear trade-off between communication efficiency and delivery time exists (see Table 1): for instance, BroadBand accounts for short delivery times (∼500 ms) but communication efficiency degrades very fast (less than 0.5 after 800 m). The best compromise appears to be offered by the NarrowBand+ setup, with an efficiency greater than 0.9 at 1600 m and delivery time of around 5.3 s, but this clearly depends on the application scenario.

**Figure 10:**
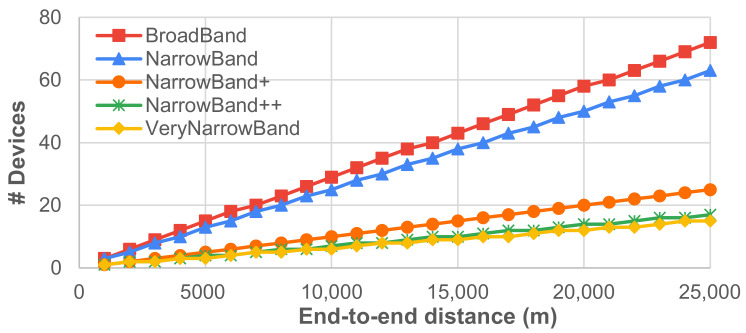
No. of intermediate nodes to ensure no-loss *multi-hop* communication.

**Figure 11:**
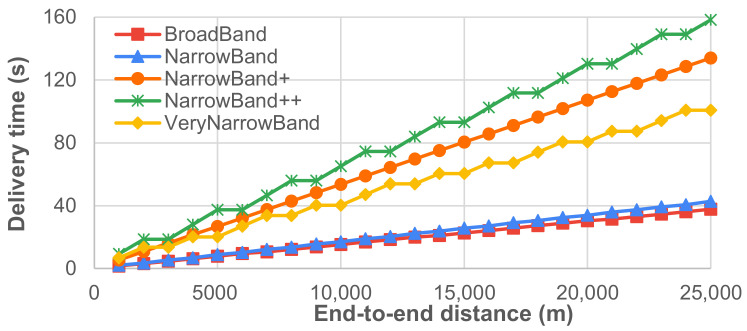
End-to-end delivery time in the case of no-loss *multi-hop* communication.

To assess the impact of multi-hop communication in data delivery, we considered the scenario where a source and a destination are far enough to be able to communicate only if their messages are routed and forwarded by a chain of intermediate nodes. We assume that intermediate nodes are always placed at the maximum distance $D_{max}$ ensuring no message loss (i.e., with efficiency equal to 1), where $D_{max}$ depends on the transmission setup and can be retrieved from Figure 9. We consider no acknowledgment and we consider, as single-hop delivery times, the values reported in Table 1, which obviously depend on the transmission setup too. Given the considerations in Section 4.2, it can be safely assumed that the end-to-end delivery time in the case of *m*-hop communication is around *m* times the single-hop delivery time for any transmission setup. Figure 10 and Figure 11 report on the expected number of intermediate nodes and end-to-end delivery time when source and destination nodes are placed at different distances. In this case, a trade-off also exists: for instance, BroadBand and NarrowBand can ensure fast communication on high end-to-end distances only if a huge number of intermediate nodes is deployed.

## 5. Conclusions and Future Work

In this paper we proposed a novel low-cost, peer-to-peer, multi-hop and gateway-free LoRa-based mesh LPWAN where the participating nodes are not just data-collecting endpoints, but can process the relatively computationally intensive applications on-site, including network-based tasks such as routing and forwarding. The proposed lightweight solution could be adopted by widely different applications requiring or benefiting from “out-of-Internet” communication, being a perfect fit for emergency scenarios. Since LoRa technology is not adapted for real time applications, the emergency scenarios we are thinking about are all those where the time constraints are comparable with the LoRa time scale. Our solution is also very flexible, since it foresees the possibility to interface the nodes (e.g., through the USB serial port) to more powerful devices directly connected to the Internet. The obtained results, although preliminary, confirm the feasibility of the approach: nodes can transmit a few parallel received/generated messages through multiple hops, and the choice of the most suitable LoRa transmission setup strictly depends on the application functional requirements.

As future work we plan to extend our experimental results to include a more extensive on-the-field evaluation of multi-hop communication, while assessing the network capabilities through some real applications. We also plan to extend the implementation to *(i)* support multiple applications on a single node and *(ii)* make the nodes interact with more complex applications running on more powerful external devices.

The following abbreviations are used in this manuscript:

## Data Availability

Not applicable.
